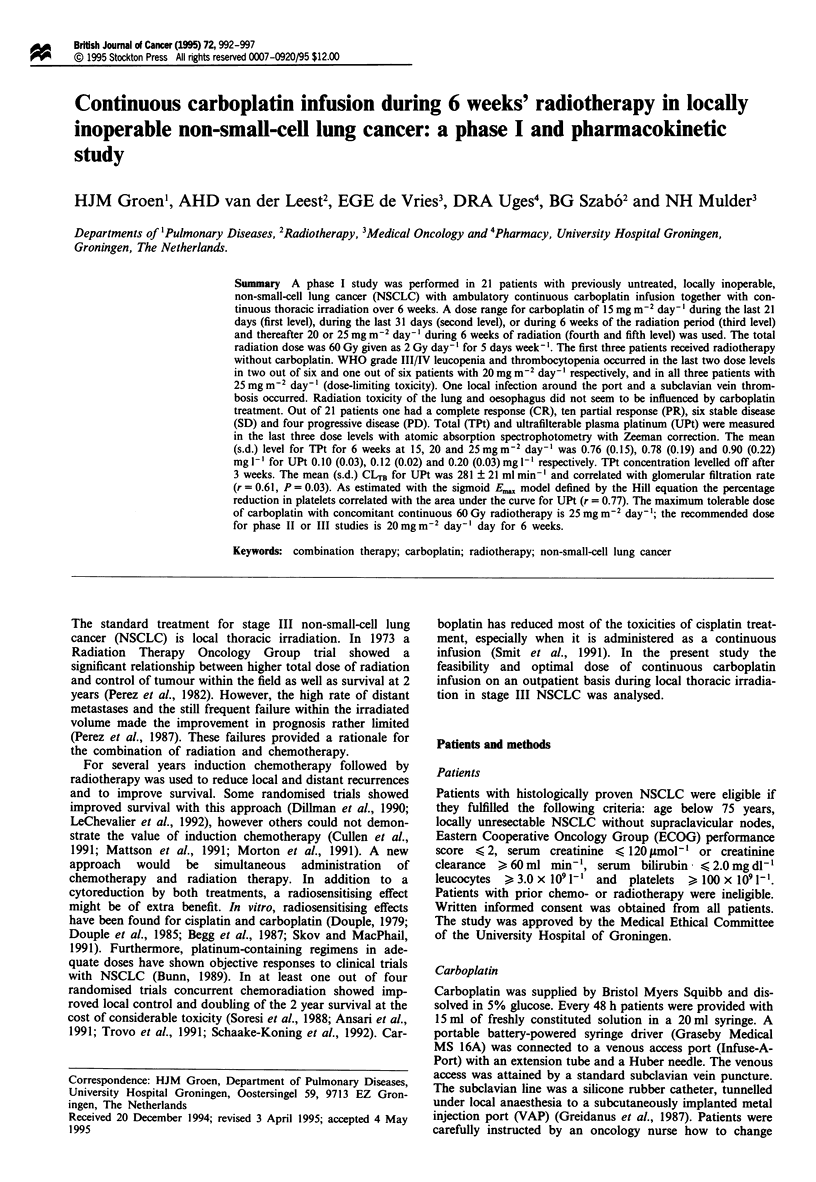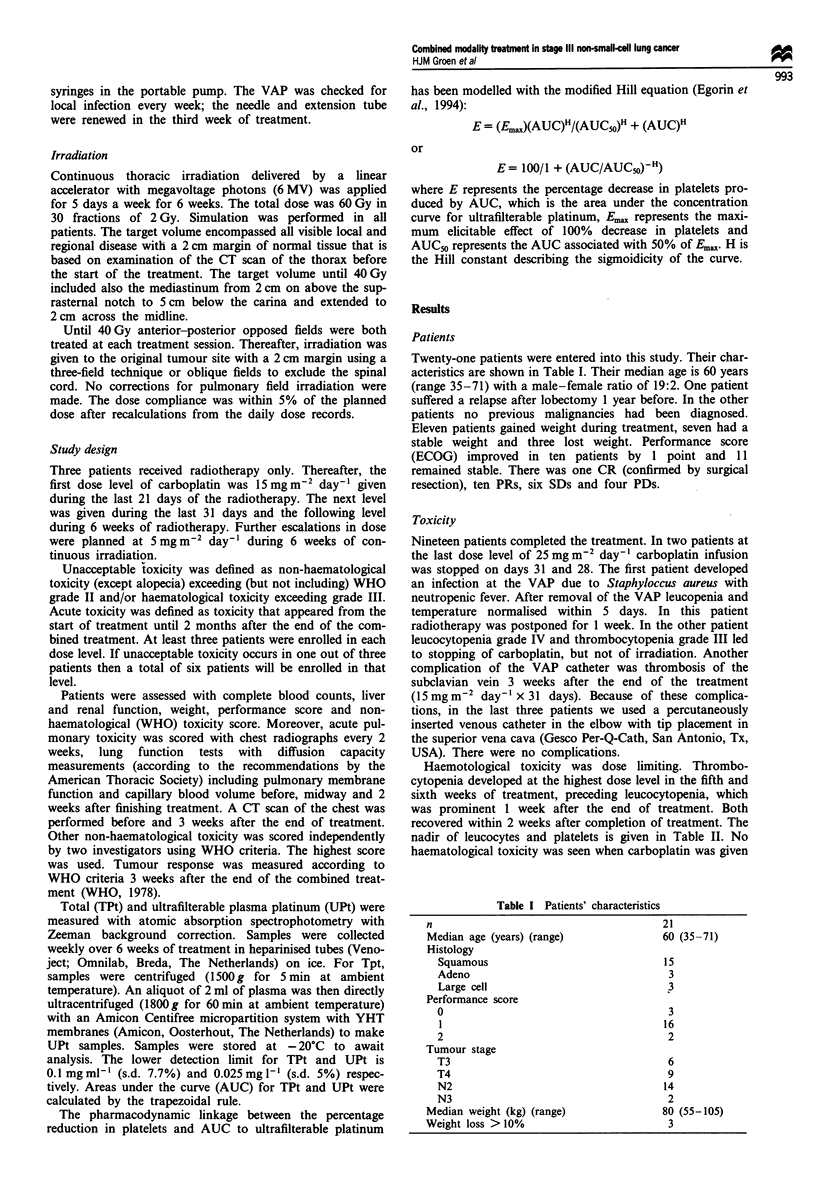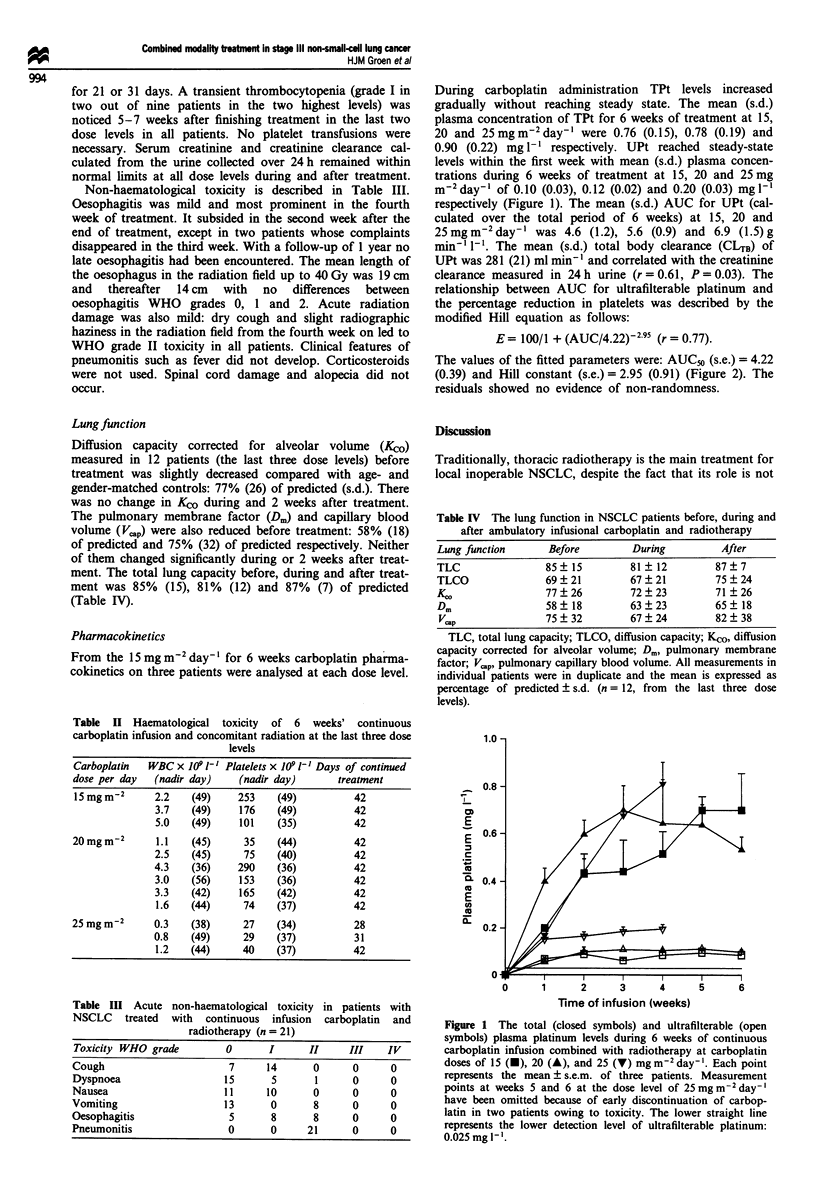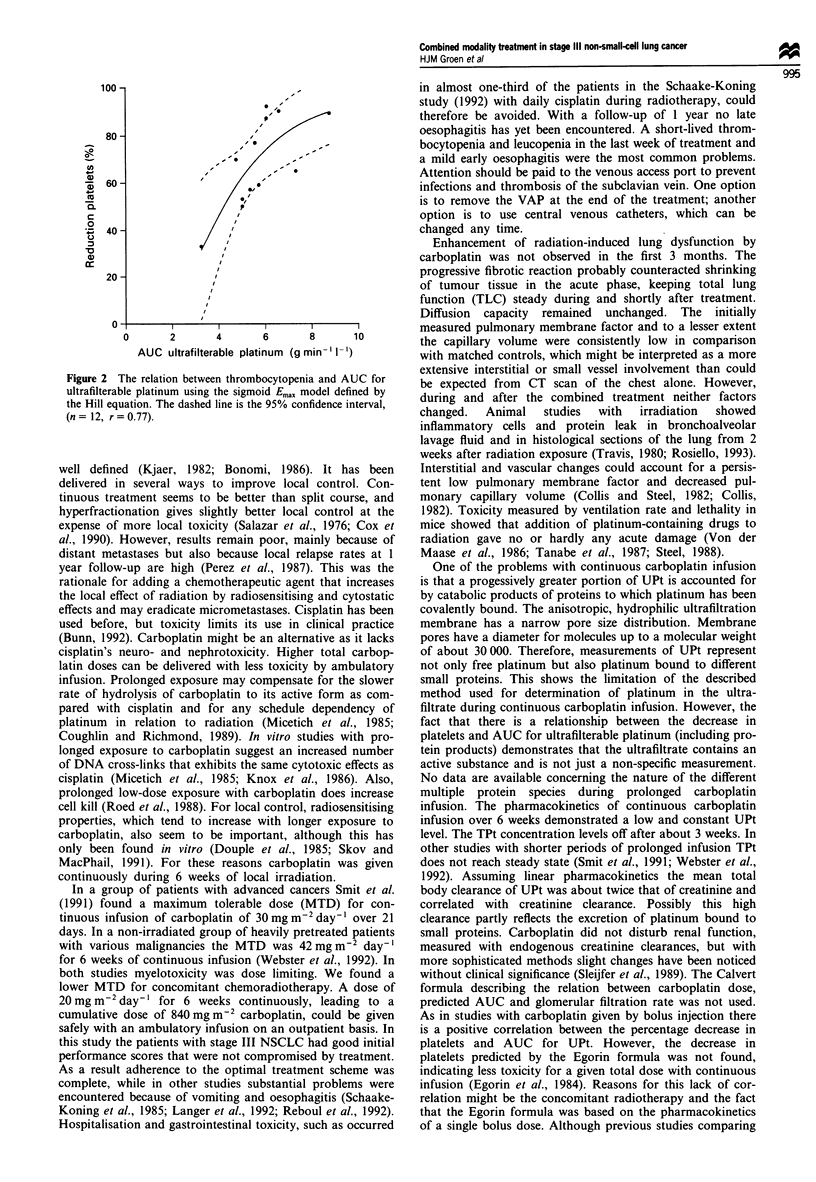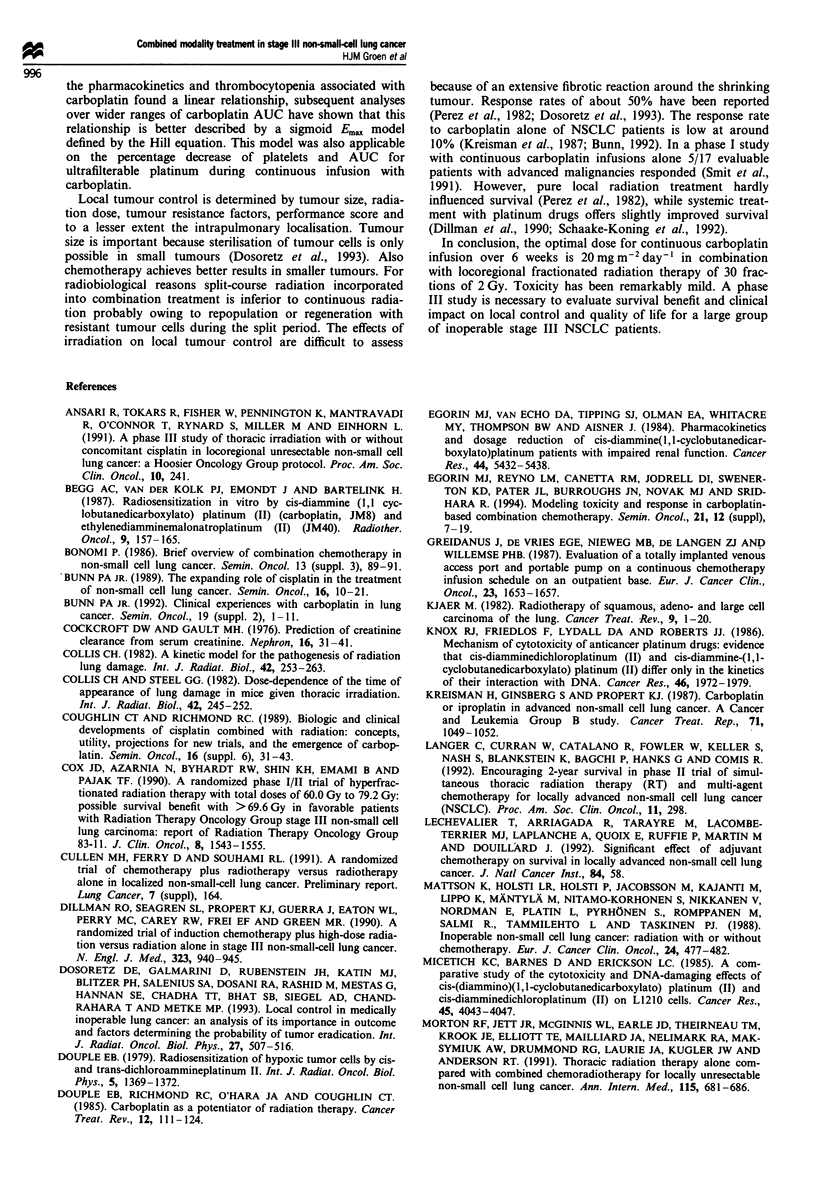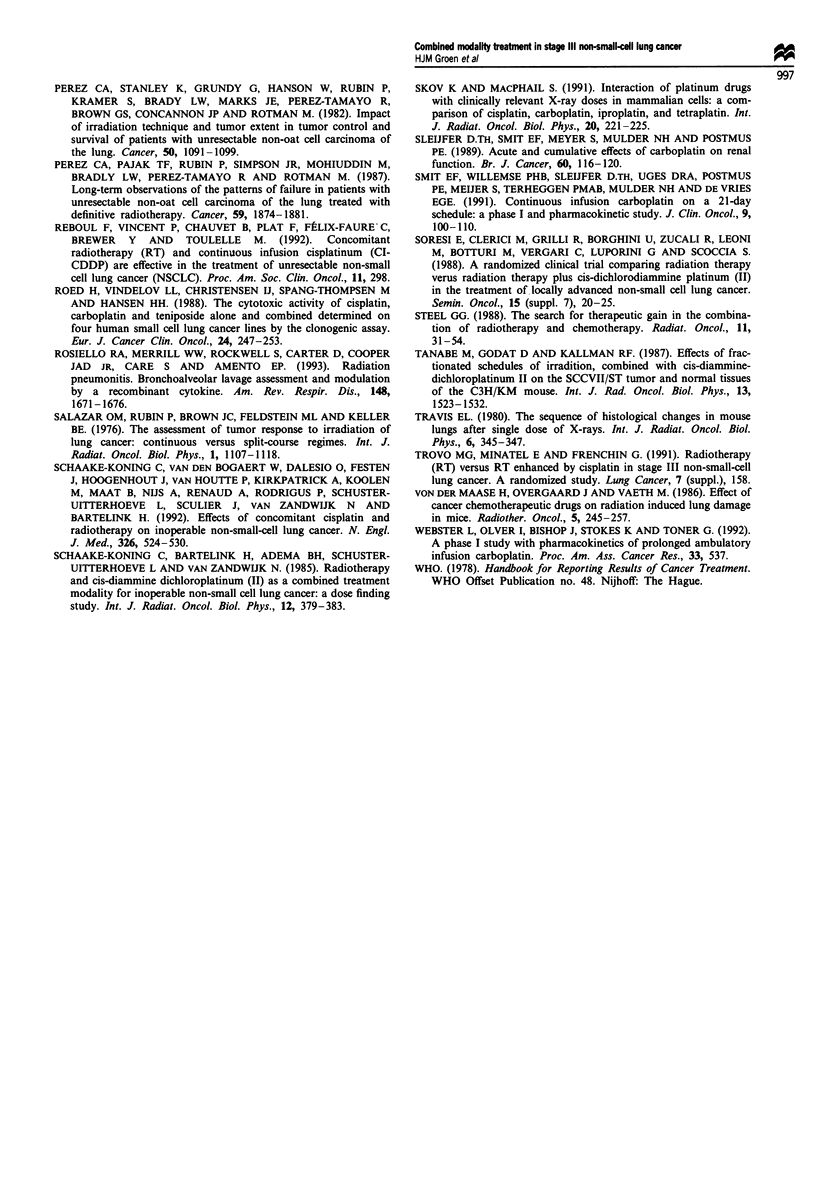# Continuous carboplatin infusion during 6 weeks' radiotherapy in locally inoperable non-small-cell lung cancer: a phase I and pharmacokinetic study.

**DOI:** 10.1038/bjc.1995.448

**Published:** 1995-10

**Authors:** H. J. Groen, A. H. van der Leest, E. G. de Vries, D. R. Uges, B. G. Szabó, N. H. Mulder

**Affiliations:** Department of Pulmonary Diseases, University Hospital Groningen, The Netherlands.

## Abstract

A phase I study was performed in 21 patients with previously untreated, locally inoperable, non-small-cell lung cancer (NSCLC) with ambulatory continuous carboplatin infusion together with continuous thoracic irradiation over 6 weeks. A dose range for carboplatin of 15 mg m-2 day-1 during the last 21 days (first level), during the last 31 days (second level), or during 6 weeks of the radiation period (third level) and thereafter 20 or 25 mg m-2 day-1 during 6 weeks of radiation (fourth and fifth level) was used. The total radiation dose was 60 Gy given as 2 Gy day-1 for 5 days week-1. The first three patients received radiotherapy without carboplatin. WHO grade III/IV leucopenia and thrombocytopenia occurred in the last two dose levels in two out of six and one out of six patients with 20 mg m-2 day-1 respectively, and in all three patients with 25 mg m-2 day-1 (dose-limiting toxicity). One local infection around the port and a subclavian vein thrombosis occurred. Radiation toxicity of the lung and oesophagus did not seem to be influenced by carboplatin treatment. Out of 21 patients one had a complete response (CR), ten partial response (PR), six stable disease (SD) and four progressive disease (PD). Total (TPt) and ultrafilterable plasma platinum (UPt) were measured in the last three dose levels with atomic absorption spectrophotometry with Zeeman correction. The mean (s.d.) level for TPt for 6 weeks at 15, 20 and 25 mg m-2 day-1 was 0.76 (0.15), 0.78 (0.19) and 0.90 (0.22) mg l-1 for UPt 0.10 (0.03), 0.12 (0.02) and 0.20 (0.03) mg l-1 respectively. TPt concentration levelled off after 3 weeks. The mean (s.d.) CLTB for UPt was 281 +/- 21 ml min-1 and correlated with glomerular filtration rate (r = 0.61, P = 0.03). As estimated with the sigmoid Emax model defined by the Hill equation the percentage reduction in platelets correlated with the area under the curve for UPt (r = 0.77). The maximum tolerable dose of carboplatin with concomitant continuous 60 Gy radiotherapy is 25 mg m-2 day-1; the recommended dose for phase II or III studies is 20 mg m-2 day-1 day for 6 weeks.


British Journal of Cancer (1995) 72, 992-997
© 1995 Stockton Press All rights reserved 0007-0920/95 $12.00

### Continuous carboplatin infusion during 6 weeks' radiotherapy in locally inoperable non-small-cell lung cancer: a phase I and pharmacokinetic study

HJM Groen[1], AHD van der Leest[2], EGE de Vries[3], DRA Uges[4], BG Szabó[2] and NH Mulder[3]

*Departments of [1]Pulmonary Diseases, [2]Radiotherapy, [3]Medical Oncology and [4]Pharmacy, University Hospital Groningen, Groningen, The Netherlands.*

**Summary** A phase I study was performed in 21 patients with previously untreated, locally inoperable, non-small-cell lung cancer (NSCLC) with ambulatory continuous carboplatin infusion together with continuous thoracic irradiation over 6 weeks. A dose range for carboplatin of 15 mg m$^{-2}$ day$^{-1}$ during the last 21 days (first level), during the last 31 days (second level), or during 6 weeks of the radiation period (third level) and thereafter 20 or 25 mg m$^{-2}$ day$^{-1}$ during 6 weeks of radiation (fourth and fifth level) was used. The total radiation dose was 60 Gy given as 2 Gy day$^{-1}$ for 5 days week$^{-1}$. The first three patients received radiotherapy without carboplatin. WHO grade III/IV leucopenia and thrombocytopenia occurred in the last two dose levels in two out of six and one out of six patients with 20 mg m$^{-2}$ day$^{-1}$ respectively, and in all three patients with 25 mg m$^{-2}$ day$^{-1}$ (dose-limiting toxicity). One local infection around the port and a subclavian vein thrombosis occurred. Radiation toxicity of the lung and oesophagus did not seem to be influenced by carboplatin treatment. Out of 21 patients one had a complete response (CR), ten partial response (PR), six stable disease (SD) and four progressive disease (PD). Total (TPt) and ultrafilterable plasma platinum (UPt) were measured in the last three dose levels with atomic absorption spectrophotometry with Zeeman correction. The mean (s.d.) level for TPt for 6 weeks at 15, 20 and 25 mg m$^{-2}$ day$^{-1}$ was 0.76 (0.15), 0.78 (0.19) and 0.90 (0.22) mg l$^{-1}$ for UPt 0.10 (0.03), 0.12 (0.02) and 0.20 (0.03) mg l$^{-1}$ respectively. TPt concentration levelled off after 3 weeks. The mean (s.d.) $CL_{TB}$ for UPt was 281 ± 21 ml min$^{-1}$ and correlated with glomerular filtration rate ($r = 0.61$, $P = 0.03$). As estimated with the sigmoid $E_{max}$ model defined by the Hill equation the percentage reduction in platelets correlated with the area under the curve for UPt ($r = 0.77$). The maximum tolerable dose of carboplatin with concomitant continuous 60 Gy radiotherapy is 25 mg m$^{-2}$ day$^{-1}$; the recommended dose for phase II or III studies is 20 mg m$^{-2}$ day$^{-1}$ day for 6 weeks.

**Keywords:** combination therapy; carboplatin; radiotherapy; non-small-cell lung cancer

The standard treatment for stage III non-small-cell lung cancer (NSCLC) is local thoracic irradiation. In 1973 a Radiation Therapy Oncology Group trial showed a significant relationship between higher total dose of radiation and control of tumour within the field as well as survival at 2 years (Perez *et al.*, 1982). However, the high rate of distant metastases and the still frequent failure within the irradiated volume made the improvement in prognosis rather limited (Perez *et al.*, 1987). These failures provided a rationale for the combination of radiation and chemotherapy.

For several years induction chemotherapy followed by radiotherapy was used to reduce local and distant recurrences and to improve survival. Some randomised trials showed improved survival with this approach (Dillman *et al.*, 1990; LeChevalier *et al.*, 1992), however others could not demonstrate the value of induction chemotherapy (Cullen *et al.*, 1991; Mattson *et al.*, 1991; Morton *et al.*, 1991). A new approach would be simultaneous administration of chemotherapy and radiation therapy. In addition to a cytoreduction by both treatments, a radiosensitising effect might be of extra benefit. *In vitro*, radiosensitising effects have been found for cisplatin and carboplatin (Douple, 1979; Douple *et al.*, 1985; Begg *et al.*, 1987; Skov and MacPhail, 1991). Furthermore, platinum-containing regimens in adequate doses have shown objective responses to clinical trials with NSCLC (Bunn, 1989). In at least one out of four randomised trials concurrent chemoradiation showed improved local control and doubling of the 2 year survival at the cost of considerable toxicity (Soresi *et al.*, 1988; Ansari *et al.*, 1991; Trovo *et al.*, 1991; Schaake-Koning *et al.*, 1992). Carboplatin has reduced most of the toxicities of cisplatin treatment, especially when it is administered as a continuous infusion (Smit *et al.*, 1991). In the present study the feasibility and optimal dose of continuous carboplatin infusion on an outpatient basis during local thoracic irradiation in stage III NSCLC was analysed.

Correspondence: HJM Groen, Department of Pulmonary Diseases, University Hospital Groningen, Oostersingel 59, 9713 EZ Groningen, The Netherlands
Received 20 December 1994; revised 3 April 1995; accepted 4 May 1995

### Patients and methods

### *Patients*

Patients with histologically proven NSCLC were eligible if they fulfilled the following criteria: age below 75 years, locally unresectable NSCLC without supraclavicular nodes, Eastern Cooperative Oncology Group (ECOG) performance score $\leqslant 2$, serum creatinine $\leqslant 120\,\mu$mol$^{-1}$ or creatinine clearance $\geqslant 60$ ml min$^{-1}$, serum bilirubin $\leqslant 2.0$ mg dl$^{-1}$ leucocytes $\geqslant 3.0 \times 10^9$ l$^{-1}$ and platelets $\geqslant 100 \times 10^9$ l$^{-1}$. Patients with prior chemo- or radiotherapy were ineligible. Written informed consent was obtained from all patients. The study was approved by the Medical Ethical Committee of the University Hospital of Groningen.

### *Carboplatin*

Carboplatin was supplied by Bristol Myers Squibb and dissolved in 5% glucose. Every 48 h patients were provided with 15 ml of freshly constituted solution in a 20 ml syringe. A portable battery-powered syringe driver (Graseby Medical MS 16A) was connected to a venous access port (Infuse-A-Port) with an extension tube and a Huber needle. The venous access was attained by a standard subclavian vein puncture. The subclavian line was a silicone rubber catheter, tunnelled under local anaesthesia to a subcutaneously implanted metal injection port (VAP) (Greidanus *et al.*, 1987). Patients were carefully instructed by an oncology nurse how to change syringes in the portable pump. The VAP was checked for local infection every week; the needle and extension tube were renewed in the third week of treatment.

*Irradiation*

Continuous thoracic irradiation delivered by a linear accelerator with megavoltage photons (6 MV) was applied for 5 days a week for 6 weeks. The total dose was 60 Gy in 30 fractions of 2 Gy. Simulation was performed in all patients. The target volume encompassed all visible local and regional disease with a 2 cm margin of normal tissue that is based on examination of the CT scan of the thorax before the start of the treatment. The target volume until 40 Gy included also the mediastinum from 2 cm on above the suprasternal notch to 5 cm below the carina and extended to 2 cm across the midline.

Until 40 Gy anterior–posterior opposed fields were both treated at each treatment session. Thereafter, irradiation was given to the original tumour site with a 2 cm margin using a three-field technique or oblique fields to exclude the spinal cord. No corrections for pulmonary field irradiation were made. The dose compliance was within 5% of the planned dose after recalculations from the daily dose records.

*Study design*

Three patients received radiotherapy only. Thereafter, the first dose level of carboplatin was 15 mg $m^{-2}$ $day^{-1}$ given during the last 21 days of the radiotherapy. The next level was given during the last 31 days and the following level during 6 weeks of radiotherapy. Further escalations in dose were planned at 5 mg $m^{-2}$ $day^{-1}$ during 6 weeks of continuous irradiation.

Unacceptable toxicity was defined as non-haematological toxicity (except alopecia) exceeding (but not including) WHO grade II and/or haematological toxicity exceeding grade III. Acute toxicity was defined as toxicity that appeared from the start of treatment until 2 months after the end of the combined treatment. At least three patients were enrolled in each dose level. If unacceptable toxicity occurs in one out of three patients then a total of six patients will be enrolled in that level.

Patients were assessed with complete blood counts, liver and renal function, weight, performance score and non-haematological (WHO) toxicity score. Moreover, acute pulmonary toxicity was scored with chest radiographs every 2 weeks, lung function tests with diffusion capacity measurements (according to the recommendations by the American Thoracic Society) including pulmonary membrane function and capillary blood volume before, midway and 2 weeks after finishing treatment. A CT scan of the chest was performed before and 3 weeks after the end of treatment. Other non-haematological toxicity was scored independently by two investigators using WHO criteria. The highest score was used. Tumour response was measured according to WHO criteria 3 weeks after the end of the combined treatment (WHO, 1978).

Total (TPt) and ultrafilterable plasma platinum (UPt) were measured with atomic absorption spectrophotometry with Zeeman background correction. Samples were collected weekly over 6 weeks of treatment in heparinised tubes (Venoject; Omnilab, Breda, The Netherlands) on ice. For Tpt, samples were centrifuged (1500 *g* for 5 min at ambient temperature). An aliquot of 2 ml of plasma was then directly ultracentrifuged (1800 *g* for 60 min at ambient temperature) with an Amicon Centifree micropartition system with YHT membranes (Amicon, Oosterhout, The Netherlands) to make UPt samples. Samples were stored at −20°C to await analysis. The lower detection limit for TPt and UPt is 0.1 mg $ml^{-1}$ (s.d. 7.7%) and 0.025 mg $l^{-1}$ (s.d. 5%) respectively. Areas under the curve (AUC) for TPt and UPt were calculated by the trapezoidal rule.

The pharmacodynamic linkage between the percentage reduction in platelets and AUC to ultrafilterable platinum has been modelled with the modified Hill equation (Egorin *et al.*, 1994):

$$E = (E_{max})(AUC)^H/(AUC_{50})^H + (AUC)^H$$

or

$$E = 100/1 + (AUC/AUC_{50})^{-H})$$

where $E$ represents the percentage decrease in platelets produced by AUC, which is the area under the concentration curve for ultrafilterable platinum, $E_{max}$ represents the maximum elicitable effect of 100% decrease in platelets and $AUC_{50}$ represents the AUC associated with 50% of $E_{max}$. H is the Hill constant describing the sigmoidicity of the curve.

### Results

*Patients*

Twenty-one patients were entered into this study. Their characteristics are shown in Table I. Their median age is 60 years (range 35–71) with a male–female ratio of 19:2. One patient suffered a relapse after lobectomy 1 year before. In the other patients no previous malignancies had been diagnosed. Eleven patients gained weight during treatment, seven had a stable weight and three lost weight. Performance score (ECOG) improved in ten patients by 1 point and 11 remained stable. There was one CR (confirmed by surgical resection), ten PRs, six SDs and four PDs.

*Toxicity*

Nineteen patients completed the treatment. In two patients at the last dose level of 25 mg $m^{-2}$ $day^{-1}$ carboplatin infusion was stopped on days 31 and 28. The first patient developed an infection at the VAP due to *Staphyloccus aureus* with neutropenic fever. After removal of the VAP leucopenia and temperature normalised within 5 days. In this patient radiotherapy was postponed for 1 week. In the other patient leucocytopenia grade IV and thrombocytopenia grade III led to stopping of carboplatin, but not of irradiation. Another complication of the VAP catheter was thrombosis of the subclavian vein 3 weeks after the end of the treatment (15 mg $m^{-2}$ $day^{-1}$ × 31 days). Because of these complications, in the last three patients we used a percutaneously inserted venous catheter in the elbow with tip placement in the superior vena cava (Gesco Per-Q-Cath, San Antonio, Tx, USA). There were no complications.

Haemotological toxicity was dose limiting. Thrombocytopenia developed at the highest dose level in the fifth and sixth weeks of treatment, preceding leucocytopenia, which was prominent 1 week after the end of treatment. Both recovered within 2 weeks after completion of treatment. The nadir of leucocytes and platelets is given in Table II. No haematological toxicity was seen when carboplatin was given

**Table I** Patients' characteristics

| | |
|---|---|
| *n* | 21 |
| Median age (years) (range) | 60 (35–71) |
| Histology | |
| Squamous | 15 |
| Adeno | 3 |
| Large cell | 3 |
| Performance score | |
| 0 | 3 |
| 1 | 16 |
| 2 | 2 |
| Tumour stage | |
| T3 | 6 |
| T4 | 9 |
| N2 | 14 |
| N3 | 2 |
| Median weight (kg) (range) | 80 (55–105) |
| Weight loss >10% | 3 |

for 21 or 31 days. A transient thrombocytopenia (grade I in two out of nine patients in the two highest levels) was noticed 5–7 weeks after finishing treatment in the last two dose levels in all patients. No platelet transfusions were necessary. Serum creatinine and creatinine clearance calculated from the urine collected over 24 h remained within normal limits at all dose levels during and after treatment.

Non-haematological toxicity is described in Table III. Oesophagitis was mild and most prominent in the fourth week of treatment. It subsided in the second week after the end of treatment, except in two patients whose complaints disappeared in the third week. With a follow-up of 1 year no late oesophagitis had been encountered. The mean length of the oesophagus in the radiation field up to 40 Gy was 19 cm and thereafter 14 cm with no differences between oesophagitis WHO grades 0, 1 and 2. Acute radiation damage was also mild: dry cough and slight radiographic haziness in the radiation field from the fourth week on led to WHO grade II toxicity in all patients. Clinical features of pneumonitis such as fever did not develop. Corticosteroids were not used. Spinal cord damage and alopecia did not occur.

### *Lung function*

Diffusion capacity corrected for alveolar volume ($K_{CO}$) measured in 12 patients (the last three dose levels) before treatment was slightly decreased compared with age- and gender-matched controls: 77% (26) of predicted (s.d.). There was no change in $K_{CO}$ during and 2 weeks after treatment. The pulmonary membrane factor ($D_m$) and capillary blood volume ($V_{cap}$) were also reduced before treatment: 58% (18) of predicted and 75% (32) of predicted respectively. Neither of them changed significantly during or 2 weeks after treatment. The total lung capacity before, during and after treatment was 85% (15), 81% (12) and 87% (7) of predicted (Table IV).

### *Pharmacokinetics*

From the 15 mg m$^{-2}$ day$^{-1}$ for 6 weeks carboplatin pharmacokinetics on three patients were analysed at each dose level. During carboplatin administration TPt levels increased gradually without reaching steady state. The mean (s.d.) plasma concentration of TPt for 6 weeks of treatment at 15, 20 and 25 mg m$^{-2}$ day$^{-1}$ were 0.76 (0.15), 0.78 (0.19) and 0.90 (0.22) mg l$^{-1}$ respectively. UPt reached steady-state levels within the first week with mean (s.d.) plasma concentrations during 6 weeks of treatment at 15, 20 and 25 mg m$^{-2}$ day$^{-1}$ of 0.10 (0.03), 0.12 (0.02) and 0.20 (0.03) mg l$^{-1}$ respectively (Figure 1). The mean (s.d.) AUC for UPt (calculated over the total period of 6 weeks) at 15, 20 and 25 mg m$^{-2}$ day$^{-1}$ was 4.6 (1.2), 5.6 (0.9) and 6.9 (1.5) g min$^{-1}$ l$^{-1}$. The mean (s.d.) total body clearance ($CL_{TB}$) of UPt was 281 (21) ml min$^{-1}$ and correlated with the creatinine clearance measured in 24 h urine ($r = 0.61$, $P = 0.03$). The relationship between AUC for ultrafilterable platinum and the percentage reduction in platelets was described by the modified Hill equation as follows:

$$E = 100/1 + (AUC/4.22)^{-2.95} \ (r = 0.77).$$

The values of the fitted parameters were: $AUC_{50}$ (s.e.) = 4.22 (0.39) and Hill constant (s.e.) = 2.95 (0.91) (Figure 2). The residuals showed no evidence of non-randomness.

**Table II** Haematological toxicity of 6 weeks' continuous carboplatin infusion and concomitant radiation at the last three dose levels

| *Carboplatin dose per day* | *WBC × 10⁹ l⁻¹ (nadir* | *day)* | *Platelets × 10⁹ l⁻¹ (nadir* | *day)* | *Days of continued treatment* |
|---|---|---|---|---|---|
| 15 mg m$^{-2}$ | 2.2 | (49) | 253 | (49) | 42 |
| | 3.7 | (49) | 176 | (49) | 42 |
| | 5.0 | (49) | 101 | (35) | 42 |
| 20 mg m$^{-2}$ | 1.1 | (45) | 35 | (44) | 42 |
| | 2.5 | (45) | 75 | (40) | 42 |
| | 4.3 | (36) | 290 | (36) | 42 |
| | 3.0 | (56) | 153 | (36) | 42 |
| | 3.3 | (42) | 165 | (42) | 42 |
| | 1.6 | (44) | 74 | (37) | 42 |
| 25 mg m$^{-2}$ | 0.3 | (38) | 27 | (34) | 28 |
| | 0.8 | (49) | 29 | (37) | 31 |
| | 1.2 | (44) | 40 | (37) | 42 |

**Table III** Acute non-haematological toxicity in patients with NSCLC treated with continuous infusion carboplatin and radiotherapy ($n = 21$)

| *Toxicity WHO grade* | *0* | *I* | *II* | *III* | *IV* |
|---|---|---|---|---|---|
| Cough | 7 | 14 | 0 | 0 | 0 |
| Dyspnoea | 15 | 5 | 1 | 0 | 0 |
| Nausea | 11 | 10 | 0 | 0 | 0 |
| Vomiting | 13 | 0 | 8 | 0 | 0 |
| Oesophagitis | 5 | 8 | 8 | 0 | 0 |
| Pneumonitis | 0 | 0 | 21 | 0 | 0 |

### Discussion

Traditionally, thoracic radiotherapy is the main treatment for local inoperable NSCLC, despite the fact that its role is not

**Table IV** The lung function in NSCLC patients before, during and after ambulatory infusional carboplatin and radiotherapy

| *Lung function* | *Before* | *During* | *After* |
|---|---|---|---|
| TLC | 85 ± 15 | 81 ± 12 | 87 ± 7 |
| TLCO | 69 ± 21 | 67 ± 21 | 75 ± 24 |
| $K_{co}$ | 77 ± 26 | 72 ± 23 | 71 ± 26 |
| $D_m$ | 58 ± 18 | 63 ± 23 | 65 ± 18 |
| $V_{cap}$ | 75 ± 32 | 67 ± 24 | 82 ± 38 |

TLC, total lung capacity; TLCO, diffusion capacity; $K_{CO}$, diffusion capacity corrected for alveolar volume; $D_m$, pulmonary membrane factor; $V_{cap}$, pulmonary capillary blood volume. All measurements in individual patients were in duplicate and the mean is expressed as percentage of predicted ± s.d. ($n = 12$, from the last three dose levels).

**Figure 1** The total (closed symbols) and ultrafilterable (open symbols) plasma platinum levels during 6 weeks of continuous carboplatin infusion combined with radiotherapy at carboplatin doses of 15 (■), 20 (▲), and 25 (▼) mg m$^{-2}$ day$^{-1}$. Each point represents the mean ± s.e.m. of three patients. Measurement points at weeks 5 and 6 at the dose level of 25 mg m$^{-2}$ day$^{-1}$ have been omitted because of early discontinuation of carboplatin in two patients owing to toxicity. The lower straight line represents the lower detection level of ultrafilterable platinum: 0.025 mg l$^{-1}$.

**Figure 2** The relation between thrombocytopenia and AUC for ultrafilterable platinum using the sigmoid $E_{max}$ model defined by the Hill equation. The dashed line is the 95% confidence interval, ($n = 12$, $r = 0.77$).

well defined (Kjaer, 1982; Bonomi, 1986). It has been delivered in several ways to improve local control. Continuous treatment seems to be better than split course, and hyperfractionation gives slightly better local control at the expense of more local toxicity (Salazar *et al.*, 1976; Cox *et al.*, 1990). However, results remain poor, mainly because of distant metastases but also because local relapse rates at 1 year follow-up are high (Perez *et al.*, 1987). This was the rationale for adding a chemotherapeutic agent that increases the local effect of radiation by radiosensitising and cytostatic effects and may eradicate micrometastases. Cisplatin has been used before, but toxicity limits its use in clinical practice (Bunn, 1992). Carboplatin might be an alternative as it lacks cisplatin's neuro- and nephrotoxicity. Higher total carboplatin doses can be delivered with less toxicity by ambulatory infusion. Prolonged exposure may compensate for the slower rate of hydrolysis of carboplatin to its active form as compared with cisplatin and for any schedule dependency of platinum in relation to radiation (Micetich *et al.*, 1985; Coughlin and Richmond, 1989). *In vitro* studies with prolonged exposure to carboplatin suggest an increased number of DNA cross-links that exhibits the same cytotoxic effects as cisplatin (Micetich *et al.*, 1985; Knox *et al.*, 1986). Also, prolonged low-dose exposure with carboplatin does increase cell kill (Roed *et al.*, 1988). For local control, radiosensitising properties, which tend to increase with longer exposure to carboplatin, also seem to be important, although this has only been found *in vitro* (Douple *et al.*, 1985; Skov and MacPhail, 1991). For these reasons carboplatin was given continuously during 6 weeks of local irradiation.

In a group of patients with advanced cancers Smit *et al.* (1991) found a maximum tolerable dose (MTD) for continuous infusion of carboplatin of 30 mg m$^{-2}$ day$^{-1}$ over 21 days. In a non-irradiated group of heavily pretreated patients with various malignancies the MTD was 42 mg m$^{-2}$ day$^{-1}$ for 6 weeks of continuous infusion (Webster *et al.*, 1992). In both studies myelotoxicity was dose limiting. We found a lower MTD for concomitant chemoradiotherapy. A dose of 20 mg m$^{-2}$ day$^{-1}$ for 6 weeks continuously, leading to a cumulative dose of 840 mg m$^{-2}$ carboplatin, could be given safely with an ambulatory infusion on an outpatient basis. In this study the patients with stage III NSCLC had good initial performance scores that were not compromised by treatment. As a result adherence to the optimal treatment scheme was complete, while in other studies substantial problems were encountered because of vomiting and oesophagitis (Schaake-Koning *et al.*, 1985; Langer *et al.*, 1992; Reboul *et al.*, 1992). Hospitalisation and gastrointestinal toxicity, such as occurred in almost one-third of the patients in the Schaake-Koning study (1992) with daily cisplatin during radiotherapy, could therefore be avoided. With a follow-up of 1 year no late oesophagitis has yet been encountered. A short-lived thrombocytopenia and leucopenia in the last week of treatment and a mild early oesophagitis were the most common problems. Attention should be paid to the venous access port to prevent infections and thrombosis of the subclavian vein. One option is to remove the VAP at the end of the treatment; another option is to use central venous catheters, which can be changed any time.

Enhancement of radiation-induced lung dysfunction by carboplatin was not observed in the first 3 months. The progressive fibrotic reaction probably counteracted shrinking of tumour tissue in the acute phase, keeping total lung function (TLC) steady during and shortly after treatment. Diffusion capacity remained unchanged. The initially measured pulmonary membrane factor and to a lesser extent the capillary volume were consistently low in comparison with matched controls, which might be interpreted as a more extensive interstitial or small vessel involvement than could be expected from CT scan of the chest alone. However, during and after the combined treatment neither factors changed. Animal studies with irradiation showed inflammatory cells and protein leak in bronchoalveolar lavage fluid and in histological sections of the lung from 2 weeks after radiation exposure (Travis, 1980; Rosiello, 1993). Interstitial and vascular changes could account for a persistent low pulmonary membrane factor and decreased pulmonary capillary volume (Collis and Steel, 1982; Collis, 1982). Toxicity measured by ventilation rate and lethality in mice showed that addition of platinum-containing drugs to radiation gave no or hardly any acute damage (Von der Maase *et al.*, 1986; Tanabe *et al.*, 1987; Steel, 1988).

One of the problems with continuous carboplatin infusion is that a progessively greater portion of UPt is accounted for by catabolic products of proteins to which platinum has been covalently bound. The anisotropic, hydrophilic ultrafiltration membrane has a narrow pore size distribution. Membrane pores have a diameter for molecules up to a molecular weight of about 30 000. Therefore, measurements of UPt represent not only free platinum but also platinum bound to different small proteins. This shows the limitation of the described method used for determination of platinum in the ultrafiltrate during continuous carboplatin infusion. However, the fact that there is a relationship between the decrease in platelets and AUC for ultrafilterable platinum (including protein products) demonstrates that the ultrafiltrate contains an active substance and is not just a non-specific measurement. No data are available concerning the nature of the different multiple protein species during prolonged carboplatin infusion. The pharmacokinetics of continuous carboplatin infusion over 6 weeks demonstrated a low and constant UPt level. The TPt concentration levels off after about 3 weeks. In other studies with shorter periods of prolonged infusion TPt does not reach steady state (Smit *et al.*, 1991; Webster *et al.*, 1992). Assuming linear pharmacokinetics the mean total body clearance of UPt was about twice that of creatinine and correlated with creatinine clearance. Possibly this high clearance partly reflects the excretion of platinum bound to small proteins. Carboplatin did not disturb renal function, measured with endogenous creatinine clearances, but with more sophisticated methods slight changes have been noticed without clinical significance (Sleijfer *et al.*, 1989). The Calvert formula describing the relation between carboplatin dose, predicted AUC and glomerular filtration rate was not used. As in studies with carboplatin given by bolus injection there is a positive correlation between the percentage decrease in platelets and AUC for UPt. However, the decrease in platelets predicted by the Egorin formula was not found, indicating less toxicity for a given total dose with continuous infusion (Egorin *et al.*, 1984). Reasons for this lack of correlation might be the concomitant radiotherapy and the fact that the Egorin formula was based on the pharmacokinetics of a single bolus dose. Although previous studies comparing the pharmacokinetics and thrombocytopenia associated with carboplatin found a linear relationship, subsequent analyses over wider ranges of carboplatin AUC have shown that this relationship is better described by a sigmoid $E_{max}$ model defined by the Hill equation. This model was also applicable on the percentage decrease of platelets and AUC for ultrafilterable platinum during continuous infusion with carboplatin.

Local tumour control is determined by tumour size, radiation dose, tumour resistance factors, performance score and to a lesser extent the intrapulmonary localisation. Tumour size is important because sterilisation of tumour cells is only possible in small tumours (Dosoretz *et al.*, 1993). Also chemotherapy achieves better results in smaller tumours. For radiobiological reasons split-course radiation incorporated into combination treatment is inferior to continuous radiation probably owing to repopulation or regeneration with resistant tumour cells during the split period. The effects of irradiation on local tumour control are difficult to assess because of an extensive fibrotic reaction around the shrinking tumour. Response rates of about 50% have been reported (Perez *et al.*, 1982; Dosoretz *et al.*, 1993). The response rate to carboplatin alone of NSCLC patients is low at around 10% (Kreisman *et al.*, 1987; Bunn, 1992). In a phase I study with continuous carboplatin infusions alone 5/17 evaluable patients with advanced malignancies responded (Smit *et al.*, 1991). However, pure local radiation treatment hardly influenced survival (Perez *et al.*, 1982), while systemic treatment with platinum drugs offers slightly improved survival (Dillman *et al.*, 1990; Schaake-Koning *et al.*, 1992).

In conclusion, the optimal dose for continuous carboplatin infusion over 6 weeks is 20 mg $m^{-2}$ $day^{-1}$ in combination with locoregional fractionated radiation therapy of 30 fractions of 2 Gy. Toxicity has been remarkably mild. A phase III study is necessary to evaluate survival benefit and clinical impact on local control and quality of life for a large group of inoperable stage III NSCLC patients.

### References

ANSARI R, TOKARS R, FISHER W, PENNINGTON K, MANTRAVADI R, O'CONNOR T, RYNARD S, MILLER M AND EINHORN L. (1991). A phase III study of thoracic irradiation with or without concomitant cisplatin in locoregional unresectable non-small cell lung cancer: a Hoosier Oncology Group protocol. *Proc. Am. Soc. Clin. Oncol.*, **10**, 241.

BEGG AC, VAN DER KOLK PJ, EMONDT J AND BARTELINK H. (1987). Radiosensitization in vitro by cis-diammine (1,1 cyclobutanedicarboxylato) platinum (II) (carboplatin, JM8) and ethylenediamminemalonatroplatinum (II) (JM40). *Radiother. Oncol.*, **9**, 157-165.

BONOMI P. (1986). Brief overview of combination chemotherapy in non-small cell lung cancer. *Semin. Oncol.* 13 (suppl. 3), 89-91.

BUNN PA JR. (1989). The expanding role of cisplatin in the treatment of non-small cell lung cancer. *Semin. Oncol.*, **16**, 10-21.

BUNN PA JR. (1992). Clinical experiences with carboplatin in lung cancer. *Semin. Oncol.*, 19 (suppl. 2), 1-11.

COCKCROFT DW AND GAULT MH. (1976). Prediction of creatinine clearance from serum creatinine. *Nephron*, **16**, 31-41.

COLLIS CH. (1982). A kinetic model for the pathogenesis of radiation lung damage. *Int. J. Radiat. Biol.*, **42**, 253-263.

COLLIS CH AND STEEL GG. (1982). Dose-dependence of the time of appearance of lung damage in mice given thoracic irradiation. *Int. J. Radiat. Biol.*, **42**, 245-252.

COUGHLIN CT AND RICHMOND RC. (1989). Biologic and clinical developments of cisplatin combined with radiation: concepts, utility, projections for new trials, and the emergence of carboplatin. *Semin. Oncol.*, **16** (suppl. 6), 31-43.

COX JD, AZARNIA N, BYHARDT RW, SHIN KH, EMAMI B AND PAJAK TF. (1990). A randomized phase I/II trial of hyperfractionated radiation therapy with total doses of 60.0 Gy to 79.2 Gy: possible survival benefit with >69.6 Gy in favorable patients with Radiation Therapy Oncology Group stage III non-small cell lung carcinoma: report of Radiation Therapy Oncology Group 83-11. *J. Clin. Oncol.*, **8**, 1543-1555.

CULLEN MH, FERRY D AND SOUHAMI RL. (1991). A randomized trial of chemotherapy plus radiotherapy versus radiotherapy alone in localized non-small-cell lung cancer. Preliminary report. *Lung Cancer*, **7** (suppl), 164.

DILLMAN RO, SEAGREN SL, PROPERT KJ, GUERRA J, EATON WL, PERRY MC, CAREY RW, FREI EF AND GREEN MR. (1990). A randomized trial of induction chemotherapy plus high-dose radiation versus radiation alone in stage III non-small-cell lung cancer. *N. Engl. J. Med.*, **323**, 940-945.

DOSORETZ DE, GALMARINI D, RUBENSTEIN JH, KATIN MJ, BLITZER PH, SALENIUS SA, DOSANI RA, RASHID M, MESTAS G, HANNAN SE, CHADHA TT, BHAT SB, SIEGEL AD, CHANDRAHARA T AND METKE MP. (1993). Local control in medically inoperable lung cancer: an analysis of its importance in outcome and factors determining the probability of tumor eradication. *Int. J. Radiat. Oncol. Biol. Phys.*, **27**, 507-516.

DOUPLE EB. (1979). Radiosensitization of hypoxic tumor cells by cis- and trans-dichloroammineplatinum II. *Int. J. Radiat. Oncol. Biol. Phys.*, **5**, 1369-1372.

DOUPLE EB, RICHMOND RC, O'HARA JA AND COUGHLIN CT. (1985). Carboplatin as a potentiator of radiation therapy. *Cancer Treat. Rev.*, **12**, 111-124.

EGORIN MJ, VAN ECHO DA, TIPPING SJ, OLMAN EA, WHITACRE MY, THOMPSON BW AND AISNER J. (1984). Pharmacokinetics and dosage reduction of cis-diammine(1,1-cyclobutanedicarboxylato)platinum patients with impaired renal function. *Cancer Res.*, **44**, 5432-5438.

EGORIN MJ, REYNO LM, CANETTA RM, JODRELL DI, SWENERTON KD, PATER JL, BURROUGHS JN, NOVAK MJ AND SRIDHARA R. (1994). Modeling toxicity and response in carboplatin-based combination chemotherapy. *Semin. Oncol.*, **21**, **12** (suppl), 7-19.

GREIDANUS J, DE VRIES EGE, NIEWEG MB, DE LANGEN ZJ AND WILLEMSE PHB. (1987). Evaluation of a totally implanted venous access port and portable pump on a continuous chemotherapy infusion schedule on an outpatient base. *Eur. J. Cancer Clin., Oncol.*, **23**, 1653-1657.

KJAER M. (1982). Radiotherapy of squamous, adeno- and large cell carcinoma of the lung. *Cancer Treat. Rev.*, **9**, 1-20.

KNOX RJ, FRIEDLOS F, LYDALL DA AND ROBERTS JJ. (1986). Mechanism of cytotoxicity of anticancer platinum drugs: evidence that cis-diamminedichloroplatinum (II) and cis-diammine-(1,1-cyclobutanedicarboxylato) platinum (II) differ only in the kinetics of their interaction with DNA. *Cancer Res.*, **46**, 1972-1979.

KREISMAN H, GINSBERG S AND PROPERT KJ. (1987). Carboplatin or iproplatin in advanced non-small cell lung cancer. A Cancer and Leukemia Group B study. *Cancer Treat. Rep.*, **71**, 1049-1052.

LANGER C, CURRAN W, CATALANO R, FOWLER W, KELLER S, NASH S, BLANKSTEIN K, BAGCHI P, HANKS G AND COMIS R. (1992). Encouraging 2-year survival in phase II trial of simultaneous thoracic radiation therapy (RT) and multi-agent chemotherapy for locally advanced non-small cell lung cancer (NSCLC). *Proc. Am. Soc. Clin. Oncol.*, **11**, 298.

LECHEVALIER T, ARRIAGADA R, TARAYRE M, LACOMBE-TERRIER MJ, LAPLANCHE A, QUOIX E, RUFFIE P, MARTIN M AND DOUILLARD J. (1992). Significant effect of adjuvant chemotherapy on survival in locally advanced non-small cell lung cancer. *J. Natl Cancer Inst.*, **84**, 58.

MATTSON K, HOLSTI LR, HOLSTI P, JACOBSSON M, KAJANTI M, LIPPO K, MÄNTYLÄ M, NITAMO-KORHONEN S, NIKKANEN V, NORDMAN E, PLATIN L, PYRHÖNEN S., ROMPPANEN M, SALMI R., TAMMILEHTO L AND TASKINEN PJ. (1988). Inoperable non-small cell lung cancer: radiation with or without chemotherapy. *Eur. J. Cancer Clin. Oncol.*, **24**, 477-482.

MICETICH KC, BARNES D AND ERICKSON LC. (1985). A comparative study of the cytotoxicity and DNA-damaging effects of cis-(diammino)(1,1-cyclobutanedicarboxylato) platinum (II) and cis-diamminedichloroplatinum (II) on L1210 cells. *Cancer Res.*, **45**, 4043-4047.

MORTON RF, JETT JR, MCGINNIS WL, EARLE JD, THEIRNEAU TM, KROOK JE, ELLIOTT TE, MAILLIARD JA, NELIMARK RA, MAKSYMIUK AW, DRUMMOND RG, LAURIE JA, KUGLER JW AND ANDERSON RT. (1991). Thoracic radiation therapy alone compared with combined chemoradiotherapy for locally unresectable non-small cell lung cancer. *Ann. Intern. Med.*, **115**, 681-686.

PEREZ CA, STANLEY K, GRUNDY G, HANSON W, RUBIN P, KRAMER S, BRADY LW, MARKS JE, PEREZ-TAMAYO R, BROWN GS, CONCANNON JP AND ROTMAN M. (1982). Impact of irradiation technique and tumor extent in tumor control and survival of patients with unresectable non-oat cell carcinoma of the lung. *Cancer*, **50**, 1091–1099.

PEREZ CA, PAJAK TF, RUBIN P, SIMPSON JR, MOHIUDDIN M, BRADLY LW, PEREZ-TAMAYO R AND ROTMAN M. (1987). Long-term observations of the patterns of failure in patients with unresectable non-oat cell carcinoma of the lung treated with definitive radiotherapy. *Cancer*, **59**, 1874–1881.

REBOUL F, VINCENT P, CHAUVET B, PLAT F, FÉLIX-FAURE C, BREWER Y AND TOULELLE M. (1992). Concomitant radiotherapy (RT) and continuous infusion cisplatinum (CI-CDDP) are effective in the treatment of unresectable non-small cell lung cancer (NSCLC). *Proc. Am. Soc. Clin. Oncol.*, **11**, 298.

ROED H, VINDELOV LL, CHRISTENSEN IJ, SPANG-THOMPSEN M AND HANSEN HH. (1988). The cytotoxic activity of cisplatin, carboplatin and teniposide alone and combined determined on four human small cell lung cancer lines by the clonogenic assay. *Eur. J. Cancer Clin. Oncol.*, **24**, 247–253.

ROSIELLO RA, MERRILL WW, ROCKWELL S, CARTER D, COOPER JAD JR, CARE S AND AMENTO EP. (1993). Radiation pneumonitis. Bronchoalveolar lavage assessment and modulation by a recombinant cytokine. *Am. Rev. Respir. Dis.*, **148**, 1671–1676.

SALAZAR OM, RUBIN P, BROWN JC, FELDSTEIN ML AND KELLER BE. (1976). The assessment of tumor response to irradiation of lung cancer: continuous versus split-course regimes. *Int. J. Radiat. Oncol. Biol. Phys.*, **1**, 1107–1118.

SCHAAKE-KONING C, VAN DEN BOGAERT W, DALESIO O, FESTEN J, HOOGENHOUT J, VAN HOUTTE P, KIRKPATRICK A, KOOLEN M, MAAT B, NIJS A, RENAUD A, RODRIGUS P, SCHUSTER-UITTERHOEVE L, SCULIER J, VAN ZANDWIJK N AND BARTELINK H. (1992). Effects of concomitant cisplatin and radiotherapy on inoperable non-small-cell lung cancer. *N. Engl. J. Med.*, **326**, 524–530.

SCHAAKE-KONING C, BARTELINK H, ADEMA BH, SCHUSTER-UITTERHOEVE L AND VAN ZANDWIJK N. (1985). Radiotherapy and cis-diammine dichloroplatinum (II) as a combined treatment modality for inoperable non-small cell lung cancer: a dose finding study. *Int. J. Radiat. Oncol. Biol. Phys.*, **12**, 379–383.

SKOV K AND MACPHAIL S. (1991). Interaction of platinum drugs with clinically relevant X-ray doses in mammalian cells: a comparison of cisplatin, carboplatin, iproplatin, and tetraplatin. *Int. J. Radiat. Oncol. Biol. Phys.*, **20**, 221–225.

SLEIJFER D.TH, SMIT EF, MEYER S, MULDER NH AND POSTMUS PE. (1989). Acute and cumulative effects of carboplatin on renal function. *Br. J. Cancer*, **60**, 116–120.

SMIT EF, WILLEMSE PHB, SLEIJFER D.TH, UGES DRA, POSTMUS PE, MEIJER S, TERHEGGEN PMAB, MULDER NH AND DE VRIES EGE. (1991). Continuous infusion carboplatin on a 21-day schedule: a phase I and pharmacokinetic study. *J. Clin. Oncol.*, **9**, 100–110.

SORESI E, CLERICI M, GRILLI R, BORGHINI U, ZUCALI R, LEONI M, BOTTURI M, VERGARI C, LUPORINI G AND SCOCCIA S. (1988). A randomized clinical trial comparing radiation therapy verus radiation therapy plus cis-dichlorodiammine platinum (II) in the treatment of locally advanced non-small cell lung cancer. *Semin. Oncol.*, **15** (suppl. 7), 20–25.

STEEL GG. (1988). The search for therapeutic gain in the combination of radiotherapy and chemotherapy. *Radiat. Oncol.*, **11**, 31–54.

TANABE M, GODAT D AND KALLMAN RF. (1987). Effects of fractionated schedules of irradition, combined with cis-diammine-dichloroplatinum II on the SCCVII/ST tumor and normal tissues of the C3H/KM mouse. *Int. J. Rad. Oncol. Biol. Phys.*, **13**, 1523–1532.

TRAVIS EL. (1980). The sequence of histological changes in mouse lungs after single dose of X-rays. *Int. J. Radiat. Oncol. Biol. Phys.*, **6**, 345–347.

TROVO MG, MINATEL E AND FRENCHIN G. (1991). Radiotherapy (RT) versus RT enhanced by cisplatin in stage III non-small-cell lung cancer. A randomized study. *Lung Cancer*, **7** (suppl.), 158.

VON DER MAASE H, OVERGAARD J AND VAETH M. (1986). Effect of cancer chemotherapeutic drugs on radiation induced lung damage in mice. *Radiother. Oncol.*, **5**, 245–257.

WEBSTER L, OLVER I, BISHOP J, STOKES K AND TONER G. (1992). A phase I study with pharmacokinetics of prolonged ambulatory infusion carboplatin. *Proc. Am. Ass. Cancer Res.*, **33**, 537.

WHO. (1978). *Handbook for Reporting Results of Cancer Treatment.* WHO Offset Publication no. 48. Nijhoff: The Hague.